# Evaluation of the Impact of a Smartphone App on Adherence to an Exercise Program in People With Chronic Low Back Pain: Randomized Controlled Trial

**DOI:** 10.2196/77736

**Published:** 2026-06-15

**Authors:** Jean-Baptiste Lechauve, Lech Dobija, Bruno Pereira, Maxime Grolier, Mathilde Pelletier-Visa, Charlotte Lanhers, Emmanuel Coudeyre

**Affiliations:** 1 Physical Medicine and Rehabilitation Department, Centre Hospitalier Universitaire de Clermont-Ferrand, Service de Médecine Physique et de Réadaptation CHU Clermont-Ferrand, 58 rue Montalembert, Clermont-Ferrand, 63003, France, 33 0672534260; 2 Direction de la recherche clinique et de l'innovation, Centre Hospitalier Universitaire de Clermont-Ferrand, Clermont-ferrand, France

**Keywords:** chronic low back pain, smartphone app, adherence, exercise, self-management

## Abstract

**Background:**

The benefits of multidisciplinary rehabilitation programs on pain and function in people with chronic low back pain (CLBP) are fairly well documented in the literature. However, these positive effects tend to fade over time due to low long-term patient adherence.

**Objective:**

The study aimed to evaluate the impact of a smartphone app on adherence to an exercise program for people with CLBP at 6 months. The secondary aims were to assess the effectiveness of the app on pain, disability, fears, and beliefs related to physical activity, physical capacity, and qualitative adherence (correctness of exercise execution) at 6 months.

**Methods:**

A total of 110 people with CLBP were included and randomized into 2 groups: 54 in the intervention group (IG) who received education on the use of the app in addition to usual care (a 3-week multidisciplinary rehabilitation program with self-management education) and 56 in the control group (CG) who received only usual care. Part B of the Exercise Adherence Rating Scale was used as the primary outcome to assess adherence to the 6-month exercise program. Secondary outcomes were pain (Numeric Rating Scale), disability (Oswestry Disability Index), barriers and facilitators to performing physical activity (Evaluation of Physical Activity Perception), physical capacity (battery of tests), and qualitative adherence (correctness of exercise execution). Statistical analyses were performed according to the intention-to-treat principle. A linear mixed model compared the primary end point between the groups at 6 months.

**Results:**

A total of 71 of 110 participants (n=35 in the CG vs n=36 in the IG) were evaluated at 6 months. We did not observe any significant difference in the Exercise Adherence Rating Scale score between the CG and the IG (group difference 0.01, 95% CI −2.4 to 2.4; *P*=.97). The same findings were found for pain, disability, and barriers and facilitators to physical activity, except for the motivation criterion. The 6-Minute Walk Test (group difference [log] 0.06, 95% CI 0.01-0.12; *P*=.06), cycle ergometer (group difference 9.30, 95% CI 0.48-18.13; *P*=.04), Ito (group difference [log] 0.31, 95% CI 0.01-0.62; *P*=.04), plank (group difference [log] 0.29, 95% CI 0.03-0.54; *P*=.03), and qualitative adherence (IG mean 12.6, SD 1.3 vs CG mean 11.4, SD 1.4; *P*=.02) differed between the groups in favor of the IG. All outcomes improved from baseline to 6 months in the IG but not in the CG.

**Conclusions:**

The smartphone app did not impact adherence to an exercise program at 6 months in individuals with CLBP. Similar results were found for pain and function. Nevertheless, the app could be a useful self-management tool in view of the positive effects on physical capacity and qualitative adherence.

## Introduction

### Background

Low back pain is the leading cause of disability worldwide, affecting over 620 million people in 2020 [1]. Although this condition typically improves in most cases, 10% to 20% of individuals continue to experience pain beyond 3 months. Pain lasting more than 3 months is classified as chronic [1]. Chronic low back pain (CLBP) leads to significant functional limitations, impacting daily activities, productivity, and overall quality of life [2]. It imposes a heavy social burden and economic cost, representing a huge challenge to health care systems. Despite its high prevalence, effective management remains a challenge.

Global health organizations, including the World Health Organization and the Global Burden of Disease Study, emphasize the importance of nonpharmacological treatments for managing CLBP. Evidence-based recommendations prioritize physical rehabilitation, including tailored exercise programs, patient education with self-care strategies, and cognitive behavioral therapy (CBT) [1-3]. Exercise is consistently recommended as a first-line treatment to improve mobility, strength, and overall function while reducing pain. Tailored exercise programs for individuals with CLBP have demonstrated significant benefits on pain and function [4]. The National Institute for Health and Care Excellence guidelines highlight the importance of self-management, providing advice tailored to individual needs to help manage CLBP over the long term [5]. Self-management programs, incorporating multidisciplinary rehabilitation, have shown positive effects on return to work and physical activity at 6 and 12 months [6]. However, maintaining the necessary level of physical activity remains a challenge for most individuals. Despite these positive results, adherence to exercise programs is often suboptimal, with dropout rates ranging from 10% to 36% [7]. Adherence is defined as “the extent to which a person’s behavior corresponds with agreed recommendations from a healthcare provider” [8]. Medium-term and long-term follow-up data show that one- to two-thirds of individuals do not comply with the exercise recommendations [9,10], particularly for unsupervised home-based exercises [11-13]. Furthermore, adherence tends to decline over time [14]. Several studies have investigated the reasons behind poor adherence to prescribed exercises following multidisciplinary rehabilitation programs. Pain and exacerbation of low back pain (LBP) are the primary barriers, negatively influencing adherence [15]. Lack of time, weather conditions [16], and difficulty integrating physical activity into daily life have also been reported as barriers [15,17]. Factors that can improve long-term adherence to a home-based exercise program include the involvement of, and follow-up by, a physiotherapist during the multidisciplinary program, as well as a clear understanding of the exercises to be performed. These facilitators improve autonomy and increase the perception of self-efficacy [18,19]. It is also a theoretical component of behavior change used in therapeutic programs [20].

CBT has emerged as a highly effective intervention for CLBP, particularly when combined with exercise [21]. CBT is “a safe, evidence-based treatment for a range of mental and physical health problems, including chronic pain” [22]. The American Pain Society and the American College of Physicians recommend combining CBT with active functional restoration exercises within a multidisciplinary management program. This combined approach can enhance adherence and reduce disability and pain over time, providing a comprehensive strategy for managing CLBP.

Connected health devices, such as smartphones and tablets, are valuable tools that can be used to deliver CBT to promote behavior change and support the sustainable adoption of healthy behaviors. These devices provide personalized medical information, self-management tools, and remote monitoring by health care professionals, creating an optimal environment for patient care. Mobile health (mHealth) solutions, particularly through smartphones, offer a means for continuous support and follow-up [23], helping maintain the benefits of in-center care through engaging, interactive platforms [24]. With the growing prevalence of smartphones (70% of the population in 2021) and the rapid expansion of health apps (from 100,000 in 2014 to 350,000 in 2021) [25], mHealth facilitates remote rehabilitation, real-time data collection, and improved disease management. As personal and accessible devices [26], smartphones enhance patient engagement and understanding of health conditions, contributing to better self-management and health care access. The use of smartphone apps could reduce pain and disability [27,28] and improve quality of life [29] in the short term, but these effects have not been studied in the long term. However, some evidence regarding the use of smartphone apps for the self-management of CLBP is contradictory [30].

### Objectives

The primary objective of this study was to assess the impact of a smartphone app on adherence to a home exercise program at 6 months in people with CLBP. The secondary aims were to assess the effectiveness of the app on pain, disability, fears, and beliefs relating to physical activity, physical capacity, and qualitative adherence (correctness of exercise execution) at 6 months.

## Methods

### Study Design

This was a single-center, prospective, controlled, cluster-randomized trial (trial registration: ClinicalTrials.gov: NCT04264949) with session as the unit of randomization. Sessions were performed 5 days per week for 3 weeks [31,32] and involved groups of 4 to 6 participants. Participants randomized to the intervention group (IG) participated in 3 one-hour sessions of education on how to use the app in addition to the conventional, multidisciplinary rehabilitation program and the self-management program. Those randomized to the control group (CG) participated only in the conventional multidisciplinary rehabilitation program and the self-management education program. Assessments were conducted at baseline, postprogram (3 wk), and at follow-up (6 mo). The trial protocol has been published elsewhere [33]. This trial is reported according to the CONSORT (Consolidated Standards of Reporting Trials) Statement for nonpharmacological trials [34] (Checklist 1).

### Recruitment and Selection

Recruitment was conducted between March 2020 and June 2023. Following a medical consultation regarding their chronic low back pain, potential participants were invited to join a multidisciplinary rehabilitation program in our physical medicine and rehabilitation department. Participants who met the inclusion criteria (Textbox 1) and provided written consent after reading the information sheet were randomized by session into one of the 2 groups.

Textbox 1. **Inclusion and exclusion criteria.**

**Inclusion criteria**

- Adult participants (≥18 years old).
- Experiencing nonspecific chronic low back pain.
- Diagnosis confirmed according to the Haute Autorité Santé definition.

Included participants must have provided written informed consent.

**Exclusion criteria**

- Participants not meeting the Haute Autorité Santé diagnostic criteria.
- Participants with comprehension difficulties (questionnaire completion impossible).
- Participants with a medical contraindication to physical exercise.
- Participants under legal guardianship, curatorship, or protective supervision.
- Participants without a smartphone.

### Randomization and Blinding

The unit of observation for the outcome was the participants. However, the session was the unit of randomization to avoid contamination bias. Individual randomization may lead to recruitment difficulties and feasibility and an increase in the number of individuals lost to follow-up in the CG. Participants in the same session were assigned to the same randomization group. An independent statistician performed the randomization and allocation using Stata v15 (StataCorp), taking into account the number of participants per session. The evaluator and the members of the rehabilitation team were blinded to group allocation. Participants were not blinded, as we had to explain the purpose of the study before randomization. The name of the app was not revealed to participants unless they were randomized into the IG or screened out of the study.

### Interventions

#### Overview

Both groups participated in a conventional, multidisciplinary rehabilitation program and a self-management education program, which lasted 3 weeks (5 days a week). The only difference between the 2 groups was that the IG received the smartphone app “Mon Coach Dos” and participated in 3 one-hour training sessions on how to use it (Multimedia Appendix 1). The aim of the app was to improve adherence to the home exercise program, reinforcing the conventional care effect.

#### Self-Management Exercise Program Based on a Smartphone App (for the IG)

The app used was Mon Coach Dos and was developed in collaboration with the health care professionals from our physical medicine and rehabilitation department and the Thuasne group. This app aims to help individuals to better understand CLBP and enable them to self-manage their condition. It provides medical information on the pathology, messages regarding the benefits of physical activity, information on pain management, and a video of a physical exercise program. It is designed to change individuals’ representations of the pathology and their related behavior [19,35]. The Mon Coach Dos app was developed following work carried out in our department on an initial test app, “E-lombactifs,” which was evaluated by collecting opinions from people with CLBP and health care professionals through interviews (individual or focus group) [23].

The main aims of the self-management exercise program using the smartphone app are to increase individuals’ understanding of (1) the importance of practicing physical exercise; (2) when, where, and how to exercise; (3) how to adapt physical activity practices according to phenotype; and (4) how to integrate these practices into daily life over the long-term. Three educational training sessions on the use of the smartphone app were provided during the rehabilitation program (each Wednesday). Each session consisted of 1 hour of self-management education and physical exercise practice related to the content of the app. By the end of the program, each participant had developed a personalized exercise program with the assistance of the adapted physical activity specialist who led the sessions.

#### Conventional, Multidisciplinary Rehabilitation Program

The rehabilitation program lasted 3 weeks. Participants attended 5 days a week (Monday to Friday). Each day included 1 hour of physical therapy, 1 hour of occupational therapy, 1 hour of adapted physical activity, 1 hour of hydrotherapy, and 1 hour of self-management education (Multimedia Appendix 2).

#### Self-Management Education

This component of the program consisted of 6 different workshops to improve the self-management of CLBP and quality of life, performed over 3 sessions (1 per week). The themes of the workshops were the anatomy and pathology of LBP, activities of daily living, nonpharmacological pain management (ie, relaxation, transcutaneous electrical nerve stimulation), pharmacological pain management, adapted physical activity, and return to work strategies.

### Outcomes

At baseline, we collected sociodemographic data (age, sex, weight, height, education status, and socioprofessional category) and medical data (history of low back pain and treatments).

#### Primary Outcome

The primary outcome was the change in the Exercise Adherence Rating Scale (EARS) score at 6 months. The EARS is a self-administered questionnaire that measures adherence to a physical activity program and has good psychometric properties [36].

#### Secondary Outcomes

Pain intensity during the last 7 days was assessed on a Numeric Rating Scale. Self-declared functional ability was measured using the Oswestry Disability Index [37,38]. Barriers and facilitators to regular physical activity were assessed using the Evaluation of Physical Activity Perception (EPAP) score [39]. Aerobic capacity was evaluated using the 6-Minute Walk Test (6 MWT) [40] and a submaximal test on a cycle ergometer [41]. Muscle endurance was evaluated using the Shirado-Ito test for the trunk flexors [42], the Sorensen test for the erector spinae [43], and the wall-sit test for the lower limbs [44]. Lumbar mobility was assessed by measuring fingertip-to-floor distance [45]. Qualitative adherence was assessed by the good execution of 3 physical exercises carried out during treatment (squat, plank, and rowing) using a graded evaluation grid out of 15 points [46]. More details are provided in Multimedia Appendix 3.

### Assessment Schedule

Study outcomes were collected at baseline, postprogram (3 wk), and at 6 months post randomization by the adapted physical activity specialist and the physician, both of whom were blinded to the group allocation.

### Statistical Analysis

The sample size estimation for this pilot trial was determined according to the 2010 CONSORT Statement extension for randomized pilot and feasibility trials [47] and Cohen recommendations [48], which define effect size (ES) limits as small (ES: 0.2), medium (ES: 0.5), and large (ES: 0.8, “grossly perceptible and therefore large”). According to data reported in the literature and considering this study as a pilot, it seemed suitable to include 60 patients per group.

To achieve an ES of 0.8 at 6 months postrandomization with a type 1 error of 5% and statistical power of 90%, 33 participants are required per group. However, because of the randomization design, with session as a unit cluster of randomization, the sample size should be increased to take into account between- and within-session variability. More precisely, the assumption in randomized controlled trials that the outcome for an individual is completely unrelated to that of any other individual is violated in cluster randomized trials because individuals in any 1 cluster (session in our case) are more likely to respond in a similar manner. This similarity is known as the intraclass correlation coefficient (ICC). For an average of 5 participants per session and an ICC of 0.05, 38 participants were required in each group. Therefore, to account for losses to follow-up, we planned to include 120 individuals (n=60 per group).

All analyses were conducted before the randomization code was broken, in line with the International Conference on Harmonization Good Clinical Practice guidelines. Data storage and management were conducted according to international guidelines relevant to French institutions. All data were entered using an electronic case report form, and data accuracy was analyzed by the study data manager. Data quality control measures included queries to identify outliers and missing data.

Continuous parameters are presented as mean (SD) or median (IQR) according to the normality of the distribution (Shapiro-Wilk test).

Participant characteristics were summarized by randomization group to consider selection biases and lack of balance.

Statistical analysis was first conducted using the intention-to-treat approach. To prevent attrition bias, missing data were replaced using post hoc multiple imputation. A linear mixed model was used to compare the primary endpoint (EARS score) between randomized groups at 6 months post randomization. The randomization group was evaluated as a fixed effect and session as a random effect to consider between- and within-session variability. The normality of residuals obtained from this model was studied. The results concerning group differences were expressed as effect sizes and 95% CIs. The estimated ICC from the fitted model was reported.

Between-group comparisons for the other outcomes were performed using random-effects models, as specified for the primary analysis.

The random-effects models were also used to study longitudinal repeated data (baseline, after rehabilitation [15 days] and at 6 months post randomization), considering participant as the random effect in addition to session. The following fixed effects were studied: randomization group, evaluation time point, and their interactions.

Statistical analyses were performed using Stata v15 (StataCorp). The tests were 2-sided with the type 1 error set at 5%. The widths of CIs were not adjusted for multiplicity and therefore should not be used in place of hypothesis testing, except for the primary outcome analysis. Consequently, the results for secondary outcomes were considered exploratory and were interpreted as such. The results were expressed using absolute group differences (after log transformation when appropriate), effect sizes, and 95% CIs.

### Ethical Considerations

This study was conducted in accordance with the ethical guidelines outlined in the Declaration of Helsinki. The study was approved by the medical ethics committee of Ile-de-France (Ile-de-France III 3740, November 5, 2019). All participants received both verbal and written information regarding the aim of the study and the protocol. Written informed consent was obtained before inclusion and before any specific procedure was performed. Participant data were anonymized and securely stored to ensure confidentiality. Identifiable information was removed from the dataset, and only deidentified data were used for analysis. Data access was restricted to authorized researchers. Participants were not compensated.

## Results

### Baseline Characteristics

**Figure 1.**
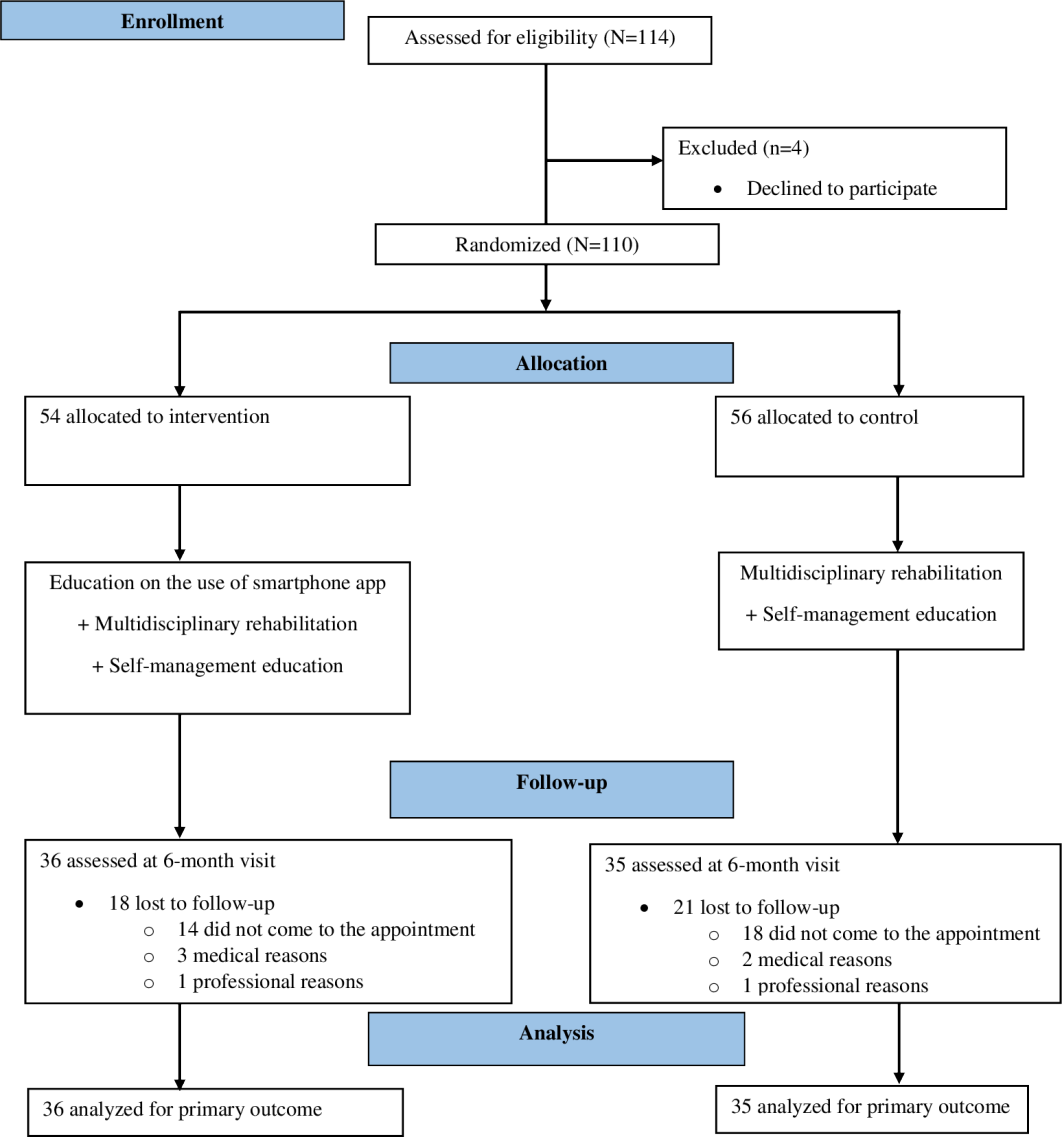
Schematic representation of the randomized controlled trial.

In total, 110 individuals were enrolled in this study (Figure 1). They were randomized either to the app-based intervention group (n=54, 49.1%) or to the control group (n=56, 50.9%). The sociodemographic and clinical characteristics of the participants at baseline are presented in Table 1.

A total of 71 (64.5%) participants completed the study. A total of 39 (35.5%) participants dropped out between “postprogram” and “M6”: 18 (46.2%) in the IG versus 21 (53.8%) in the CG.

**Table 1.** Baseline demographic and clinical characteristics of the trial groups (N=110).

Characteristics	Intervention group (n=54)	Control group (n=56)
Age (y), mean (SD)	44.6 (9.2)	45.6 (9.7)
Gender, n (%)		
Female	24 (44.4)	23 (41.1)
Male	30 (55.6)	33 (58.9)
BMI (kg/m2), mean (SD)	26.8 (4.4)	26.9 (5.6)
Work situation, n (%)		
Active	1 (1.8)	3 (5.3)
Sick leave	48 (88.9)	50 (89.3)
Disability	3 (5.6)	2 (3.6)
Retired	2 (3.7)	0 (0)
Unemployed	0 (0)	1 (1.8)
Duration of sick leave (wk), mean (SD)	36.5 (53.5)	53.2 (58.1)
Duration of low back pain (wk), mean (SD)	369.3 (383.2)	251.6 (307)
Physiotherapy treatment: Yes, n (%)	46 (85.2)	53 (94.6)
EARS^a^, mean (SD)	14.9 (6)	15.5 (5.4)
NRS^b^, mean (SD)	44.4 (23.4)	49.3 (20.6)
ODI^c^, mean (SD)	32.3 (12)	35.2 (14.2)
EPAP^d^, mean (SD)	280.1 (84.6)	279.4 (81)
6MWT^e^ (m), mean (SD)	473.6 (91.8)	477.6 (95.1)
Ergocycle test (W), mean (SD)	100.3 (28.4)	101.3 (31.2)
Ito (s), mean (SD)	63.7 (48.5)	81.8 (46.1)
Sorensen (s), mean (SD)	56.5 (41.5)	55.6 (39.3)
Wall-sit test (s), mean (SD)	60.36 (34.67)	51.34 (29.66)
Plank (s), mean (SD)	56.2 (35)	53.8 (30.2)
Fingertip-to-floor (cm), mean (SD)	18.2 (13.2)	19.9 (12.2)

a EARS: Exercise Adherence Rating Scale.

b NRS: Numeric Rating Scale.

c ODI: Oswestry Disability Index.

d EPAP: Evaluation of Physical Activity Perception.

e 6MWT: 6-Minute Walk Test.

### Primary Outcome

**Figure 2.**
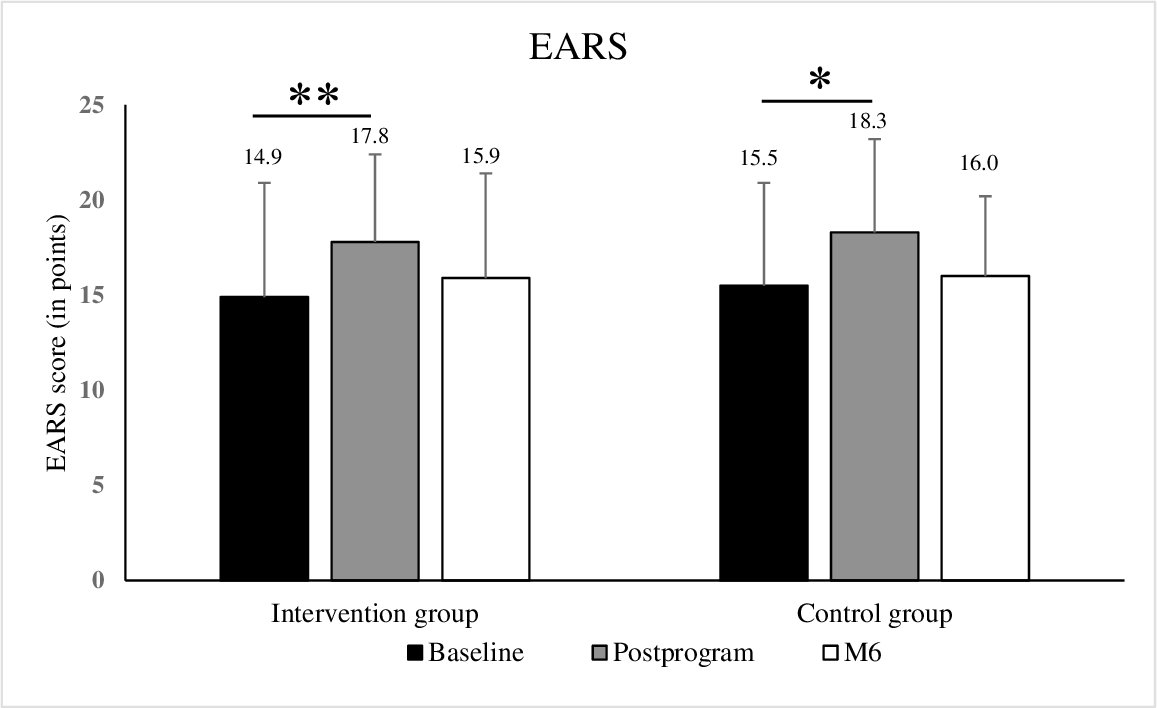
Exercise Adherence Rating Scale (EARS) score in the 2 groups at baseline, postprogram, and at 6 months. M6: 6 months. *P≤.05 and **P≤.01.

The EARS score did not differ significantly between the 2 groups at 6 months (group difference 0.01, 95% CI −2.4 to 2.4; *P*=.97). However, the EARS score improved significantly in both groups postprogram compared to baseline (Figure 2). These results were confirmed by the analysis that took into account the session effect as a random effect (group difference 0.1, 95% CI −2.4 to 2.4; *P*=.97). The ICC related to the session effect was 2.7%.

### Secondary Outcomes

All secondary outcomes are presented in Table 2.

**Table 2.** Outcomes at baseline, postprogram (3 wk), and at 6 months (M6).

Outcomes	Control group (n=35), mean (SD)	*P* value	Intervention group (n=36), mean (SD)	*P* value	Between-group, effect size (95% CI)	*P* value
EARS^a^						
Baseline	15.5 (5.4)	—^b^	14.9 (6)	—	—	—
Postprogram	18.3 (4.9)	.002	17.8 (4.6)	<.001	0.03 (−0.24 to 0.30)	.82
M6	16 (4.2)	.55	15.9 (5.46)	.55	0.00 (−0.29 to 0.30)	.97
6MWT^c^						
Baseline	477.6 (95.1)	—	473.6 (91.8)	—	—	—
Postprogram	527.3 (87.7)	<.001	555 (91.3)	<.001	0.32 (0.05 to 0.59)	.02
M6	500.8 (102.1)	<.001	545.11 (107)	<.001	0.28 (−0.01 to 0.58)	.06
Ergocycle						
Baseline	101.3 (31.2)	—	100.3 (28.4)	—	—	—
Postprogram	105.2 (32.8)	.19	110.1 (29.4)	<.001	0.22 (−0.05 to 0.49)	.11
M6	95.6 (34)	.14	107.7 (35.1)	.15	0.31 (0.02 to 0.61)	.04
Ito						
Baseline	81.8 (46.1)	—	63.7 (48.5)	—	—	—
Postprogram	98.4 (53.2)	.005	90.1 (53.7)	<.001	0.19 (−0.09 to 0.46)	.18
M6	88.8 (52.3)	.51	89.1 (52.4)	<.001	0.30 (−0.01 to 0.60)	.045
Sorensen						
Baseline	55.6 (39.3)	—	56.5 (41.5)	—	—	—
Postprogram	75.7 (47.4)	.005	85.9 (43.9)	<.001	0.22 (−0.06 to 0.49)	.12
M6	57.42 (40.2)	.43	75 (37.4)	.001	0.23 (−0.07 to 0.53)	.13
Wall-sit test						
Baseline	51.34 (29.66)	—	60.36 (34.67)	—	—	—
Postprogram	72.43 (42.91)	.001	80.81 (52.16)	.006	−0.09 (−0.36 to 0.18)	.50
M6	63.74 (39.84)	.006	77.11 (44.23)	.005	0.08 (−0.22 to 0.38)	.59
Plank						
Baseline	53.8 (30.2)	—	56.2 (35)	—	—	—
Postprogram	76.4 (38.4)	<.001	88.4 (48.7)	<.001	0.16 (−0.11 to 0.43)	.25
M6	65 (44.4)	.56	71.9 (45.1)	<.001	0.33 (0.04 to 0.63)	.03
FFD^d^						
Baseline	19.9 (12.2)	—	18.2 (13.2)	—	—	—
Postprogram	14.3 (12.2)	<.001	10.6 (12.4)	<.001	−0.17 (−0.44 to 0.10)	.22
M6	14 (11.8)	<.001	11.1 (13)	<.001	−0.01 (−0.31 to 0.28)	.93
Pain (NRS^e^)						
Baseline	49.3 (20.6)	—	44.4 (23.4)	—	—	—
Postprogram	45.5 (21.4)	.08	38.2 (22.5)	.02	−0.10 (−0.37 to 0.17)	.47
M6	48.1 (21.6)	.92	38.3 (21.6)	.03	−0.25 (−0.55 to 0.05)	.10
ODI^f^						
Baseline	35.2 (14.2)	—	32.3 (12)	—	—	—
Postprogram	31.7 (14.3)	.005	27 (14.6)	<.001	−0.15 (−0.43 to 0.12)	.27
M6	28.6 (14.8)	<.001	23 (12)	<.001	−0.16 (−0.46 to 0.14)	.29
EPAP motivation						
Baseline	84.8 (20.2)	—	79.7 (20.6)	—	—	—
Postprogram	87.1 (17.1)	.37	85.9 (20.4)	.001	0.16 (−0.11 to 0.43)	.24
M6	82.1 (22)	.52	88.9 (15.3)	.003	0.34 (0.05 to 0.64)	.02
EPAP beliefs						
Baseline	50.3 (24.1)	—	58 (24.2)	—	—	—
Postprogram	62.7 (25.7)	<.001	69.7 (26.1)	<.001	−0.02 (−0.29 to 0.25)	.89
M6	63.8 (24.6)	.001	79.2 (19)	<.001	0.13 (−0.17 to 0.42)	.41
EPAP levers						
Baseline	69.8 (18.3)	—	74.7 (16.5)	—	—	—
Postprogram	70.9 (20.3)	.72	79.7 (15.5)	<.001	0.16 (−0.11 to 0.44)	.24
M6	67.4 (18.6)	.49	78.8 (15.4)	.02	0.25 (−0.05 to 0.55)	.10
EPAP barriers						
Baseline	74.5 (18.4)	—	67.7 (23.3)	—	—	—
Postprogram	76.7 (18.1)	.39	77.2 (22)	<.001	0.40 (0.13 to 0.67)	.004
M6	76 (18.9)	.37	77.4 (21.9)	.01	0.16 (−0.13 to 0.46)	.28

a EARS: Exercise Adherence Rating Scale.

b Not applicable.

c 6MWT: 6-Minute Walk Test.

d FFD: fingertip-to-floor distance.

e NRS: Numeric Rating Scale.

f ODI: Oswestry Disability Index.

#### Pain (Numeric Rating Scale)

**Figure 3.**
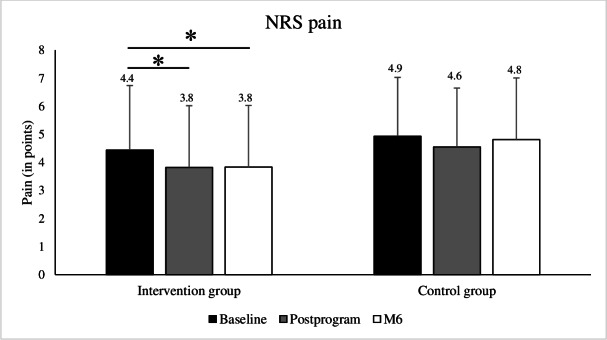
Pain score in the 2 groups at baseline, postprogram, and at 6 months. M6: 6 months; NRS: Numeric Rating Scale. *P≤.05.

Pain levels did not differ between the 2 groups at 6 months (group difference −6.58, 95% CI −14.46 to 1.29; *P*=.10). Pain reduced significantly in the IG postprogram compared to baseline (mean 3.8, SD 2.3 vs mean 4.4, SD 2.3; *P*=.02), as well as at 6 months compared to baseline (mean 3.8, SD 2.2 vs mean 4.4, SD 2.3; *P*=.03) (Figure 3). These differences were not found in the CG.

#### Disability (ODI)

Disability levels did not differ between the 2 groups at 6 months (group difference −2.27, 95% CI −6.50 to 1.96; *P*=.29). However, the ODI score improved significantly in both groups postprogram and at 6 months compared to baseline.

#### Barriers and Facilitators to Regular Physical Activity (EPAP)

The EPAP scores did not differ between the 2 groups at 6 months, except for the criterion “motivation” (group difference 8.38, 95% CI 1.12-15.65; *P*=.02). All EPAP criteria improved significantly (motivation, beliefs, barriers, facilitators) in the IG between baseline and 6 months. These differences were not found in the CG, except for the belief criterion.

#### Physical Capacity

At 6 months, there was a small between-group difference in the 6MWT (group difference [log] 0.06, 95% CI 0.01-0.12; *P*=.06), the cycle ergometer test (group difference 9.30, 95% CI 0.48-18.13; *P*=.04), the Ito (group difference [log] 0.31, 95% CI 0.01-0.62; *P*=.04), and the plank test (group difference [log] 0.29, 95% CI 0.03-0.54; *P*=.03) in favor of the IG. Moreover, a significant improvement in all physical capacity tests was found postprogram compared to baseline in both groups, except for the cycle ergometer test in the CG. This significant improvement was maintained at 6 months in the IG for all tests, except for the cycle ergometer test, whereas in the CG, only the 6-MWT, wall-sit test, and the fingertip-to-floor distance test showed an improvement at 6 months compared to baseline (Table 2).

#### Qualitative Adherence

**Figure 4.**
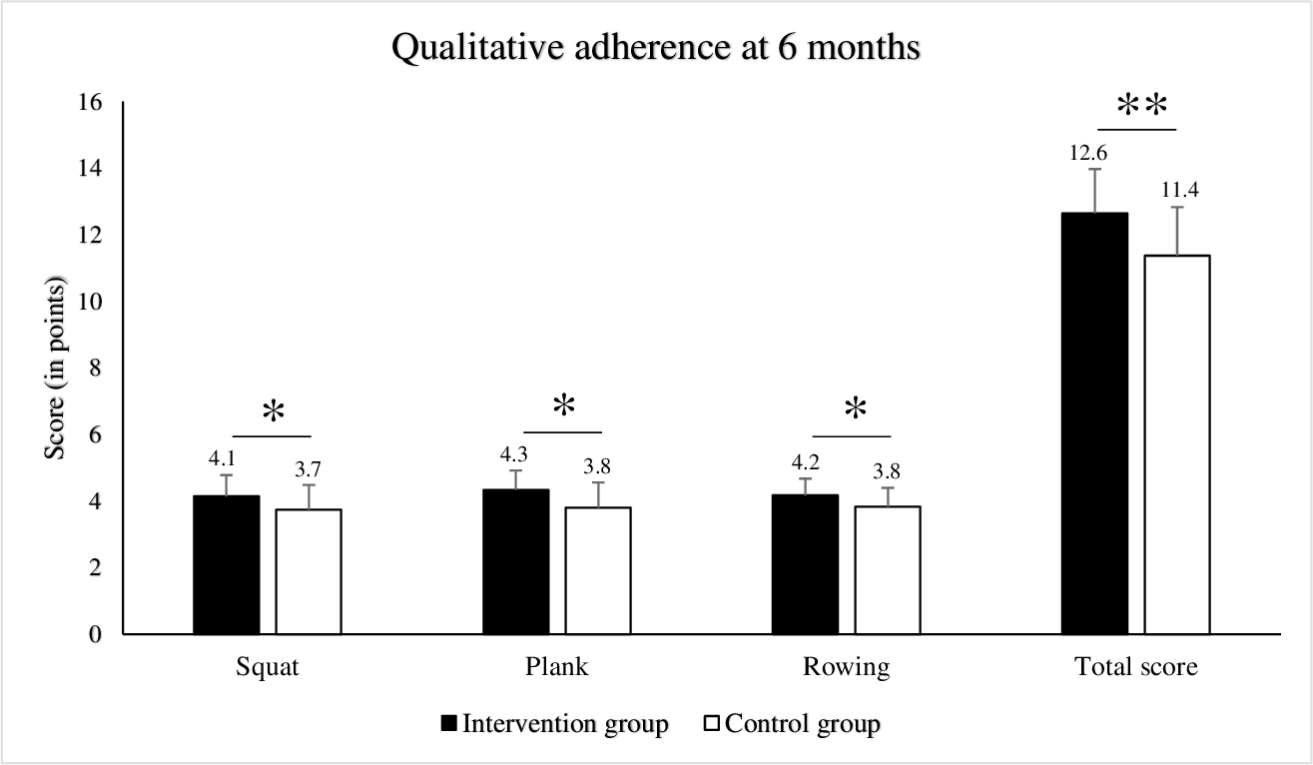
Qualitative adherence at 6 months in the 2 groups. **P≤.01, *P≤.05.

The total qualitative adherence score differed significantly between the groups in favor of the IG at 6 months (IG: 12.6, SD 1.3 vs CG: 11.4, SD 1.4; *P*=.02; Figure 4).

## Discussion

To our knowledge, this is the first study to evaluate the impact of a smartphone app on adherence to a home exercise program at 6 months in people with CLBP. The lack of a significant difference in adherence between the 2 groups at 6 months suggests that the Mon Coach Dos app did not enhance adherence compared to usual care.

### Primary Outcome

Although adherence did not differ between the groups, it improved significantly postprogram in both groups, with a return to the initial score at 6 months in both groups. This aligns with previous findings on medium- and long-term adherence, demonstrating a decline in adherence levels 3 months postprogram [49,50]. However, the study by Krkoska et al [51] showed very good adherence to a home exercise program over a period of 18 weeks. In that study, 25 out of 27 participants completed the program. Adherence was measured by the frequency of completing the program (once or twice a day) and was greater in the app group than in the diary group. However, the strong adherence of both groups to the programs can be explained by the considerable number of monitoring visits carried out during the program, making it possible to maintain a strong relationship with the therapist. In a qualitative study collecting patient expectations regarding mHealth, Palazzo et al [24] found that adherence could be increased by maintaining a relationship with the provider, for example, via telephone feedback, by offering attractive exercise programs, and by improving patient performance. In our study, we did not provide feedback to the participants during the 6-month follow-up period.

Adherence is a concept that is quite difficult to define and evaluate. Adherence to mHealth interventions has been assessed by different methods and defined in various ways. For example, some studies defined adherence as the number of complete active days of app use [52,53], the average time spent using the app [54], or the number of self-management plans completed using the app during the first 12 weeks after randomization [55]. In 2013, Beinart et al [10] highlighted the lack of standardized measures for adherence to prescribed home exercises. The absence of a standardized and valid tool to quantify adherence could compromise research on the effectiveness of tools designed to improve adherence [56]. There is currently no “gold standard” [57] nor a consensus for measuring patient adherence to self-exercise programs [58]. The development of a validated measure of adherence should be a priority for the research community, as it could lead to a better understanding of the multitude of factors that affect adherence to home exercises in people with LBP [10]. Furthermore, adherence to an exercise program can be influenced by the patient’s perception and motivation and the therapist’s expectations, which further complicates its objective measurement [59]. In response to this observation, Newman-Beinart et al [36] developed the first self-administered questionnaire to assess adherence to a physical activity program, the EARS questionnaire. This questionnaire has been used and evaluated in several languages (eg, Brazilian, Danish, Nepali) and across various pathologies, and it has been shown to have good reliability and validity [60-62]. This is why we used the French version of the EARS questionnaire to measure our primary outcome. We used Part B of the questionnaire, which quantitatively measures adherence to the exercise program. Contrary to our expectation, there was no between-group difference in the EARS score. However, this scale has already been questioned by Arensman et al [63], who stated that the EARS only focuses on the quantitative properties of adherence and not on the quality of performance of the home-based exercise program pre-established by the health care professional. For this reason, they are now developing an instrument to measure adherence in terms of frequency, intensity, and quality of performance of home-based exercise programs [63]. In our study, we were not able to collect these quantitative data (log-ins, video views, exercise completions) due to a technical problem with the app on data recovery.

### Secondary Outcomes

#### Pain or Disability

Neither pain nor disability levels differed between the groups at 6 months, in contrast with the findings of Chaabra et al [27]. A systematic review found that the simultaneous use of mHealth and usual care interventions was more effective than usual care in reducing pain intensity and disability in people with LBP [64]. Furthermore, they found that telephone calls enhanced improvements in pain and disability, suggesting that telephone calls or the provision of sensitive feedback devices enhance the effectiveness of mHealth interventions on pain intensity and disability. They could improve patient adherence and self-management [24,51]. We found a significant, long-term (6 mo) reduction in pain and disability score in the IG, a finding that has been previously reported for the short term (3 mo) [27,28,65]. The lack of a between-group difference in pain despite the significant reduction in pain at 6 months only in the IG and not in the CG could be explained by the fact that the CG received an intervention (a 3-week multidisciplinary program, including a therapeutic patient education program). In addition, we did not provide feedback or telephone calls to the IG. A scoping review found similar results regarding the effectiveness of mHealth apps on pain and disability but concluded that the generalizability of the results was limited due to the heterogeneity of the characterization of pain by the included participants and the different intervention durations [66].

#### Barriers and Facilitators to Regular Physical Activity (EPAP)

We found no difference between the groups in the EPAP criteria at 6 months, except for the “motivation” criterion, which could be explained by the fact that the smartphone app included videos of the exercises. However, all EPAP criteria (increased facilitators and decreased barriers) improved at 6 months in the IG, but not in the CG. Therefore, access to this app and its content appeared to help dispel false beliefs about physical activity and LBP. Participants might have been better informed about their pathology and gained a more precise understanding of its mechanisms. This process of clarification reduces the barriers often associated with low back pain and physical exercise [15].

#### Physical Capacity

Few studies have evaluated the long-term effectiveness of health apps on physical capacity. Our study found a significant improvement in all physical parameters at 6 months in the IG, but not in the CG, except for the 6MWT and FFT. Moreover, the significant between-group differences at 6 months in the 6MWT, cycle ergometer, Ito, and plank tests highlight the positive impact of the app on maintaining physical capacity in the long term. The lack of significant improvement in the CG, except for the specific tests mentioned, may also suggest that the mHealth app provided added value in terms of motivation or organization of exercise practice [24], facilitating progress in the IG.

#### Qualitative Adherence

The IG group executed the exercises (squat, plank, rowing) more correctly than the CG at 6 months. This could be attributed to the videos provided in the app that demonstrated the correct movements to perform. These videos offered participants a reassuring visual model, helping them gain confidence in their ability to perform the exercises correctly, which could have previously been a barrier to exercise practice. Therefore, the app appears to be a useful, motivational tool to promote continued physical activity, as its dynamic content instills user confidence in the correct execution of the movements performed, reducing the fear of injury and exacerbation of pain [15]. The app also allows users to be more independent in performing the movements, potentially leading to better qualitative adherence [67]. The qualitative aspect of adherence is important to consider when defining and evaluating adherence, in alignment with recommendations regarding the quality of exercise performance [63].

### Relationship Between Adherence and Secondary Outcomes

It is surprising that, although quantitative adherence at 6 months (EARS) did not differ between the groups, the secondary outcomes (pain, disability, and physical capacity) only improved in the IG. Another study found no association between adherence to home-based exercise recommendations and changes in clinical outcomes in people with LBP [68]. These findings suggest that the relationship between home-based exercise adherence and treatment outcomes is complex. Further research is needed to better understand this relationship.

### Limitations

The primary limitation of our study, given the existing literature, was the lack of quantitative data on the use of the app by the users (number, frequency, duration of connections to the app), which would have allowed us to be more precise in terms of adherence and to correlate these data with the EARS score.

The metrological properties of the EARS appear limited as only 6 questions were used to assess adherence [36]. Moreover, some of these questions appear to be similar. Furthermore, the EARS does not evaluate qualitative adherence.

Furthermore, our control group did not represent a true nonintervention, as the participants in the CG were provided with a conventional program, along with a patient education program, which simultaneously offered important information to help patients better manage their condition on a daily basis. The control group was not a “true” control, as seen in the previously cited studies.

Finally, although the number of dropouts during the protocol (39/110) indicates some challenges in sustaining participant engagement over a long-term process [65], the impact on the study’s statistical power is likely limited. First, in the final analysis, the observed ICC was 0.027, lower than the assumed 0.05, suggesting that the inflation applied in the sample size calculation was conservative. Moreover, the initial sample size calculation indicated that 38 participants per group were required. After inflating this number to account for potential missing data, a target of 60 participants per group was set. Although the final analysis included 35 versus 36 participants, this remains reasonably close to the initial requirement and is therefore unlikely to have markedly compromised statistical power.

### Conclusions

The Mon Coach Dos smartphone app did not improve adherence to an exercise program at 6 months in individuals with CLBP. Similarly, it did not lead to improved outcomes in pain, function, and physical capacity at 6 months. However, the significant improvement in pain, function, and physical parameters between baseline and 6 months in the IG, as well as better qualitative adherence, highlights the usefulness of such a tool in empowering individuals in the daily self-management of CLBP.

## Supplementary material

10.2196/77736 Multimedia Appendix 1 Study design.

10.2196/77736 Multimedia Appendix 2 Multidisciplinary rehabilitation program.

10.2196/77736 Multimedia Appendix 3 Graded evaluation grid of qualitative adherence.

10.2196/77736 Checklist 1 CONSORT checklist.

## Abbreviations

6MWT: 6-Minute Walk Test
CBT: cognitive behavioral therapy
CG: control group
CLBP: chronic low back pain
CONSORT: Consolidated Standards of Reporting Trials
EARS: Exercise Adherence Rating Scale
EPAP: Evaluation of Physical Activity Perception
ES: effect size
ICC: intraclass correlation coefficient
IG: intervention group
LBP: low back pain
mHealth: mobile health
