# Supplementary figures and images for: Evaluation of the Impact of a Smartphone App on Adherence to an Exercise Program in People With Chronic Low Back Pain: Randomized Controlled Trial
Source: JMIR Mhealth Uhealth. 2026 Jun 15;14:e77736. doi: 10.2196/77736 (PMC13268638; doi:10.2196/77736)

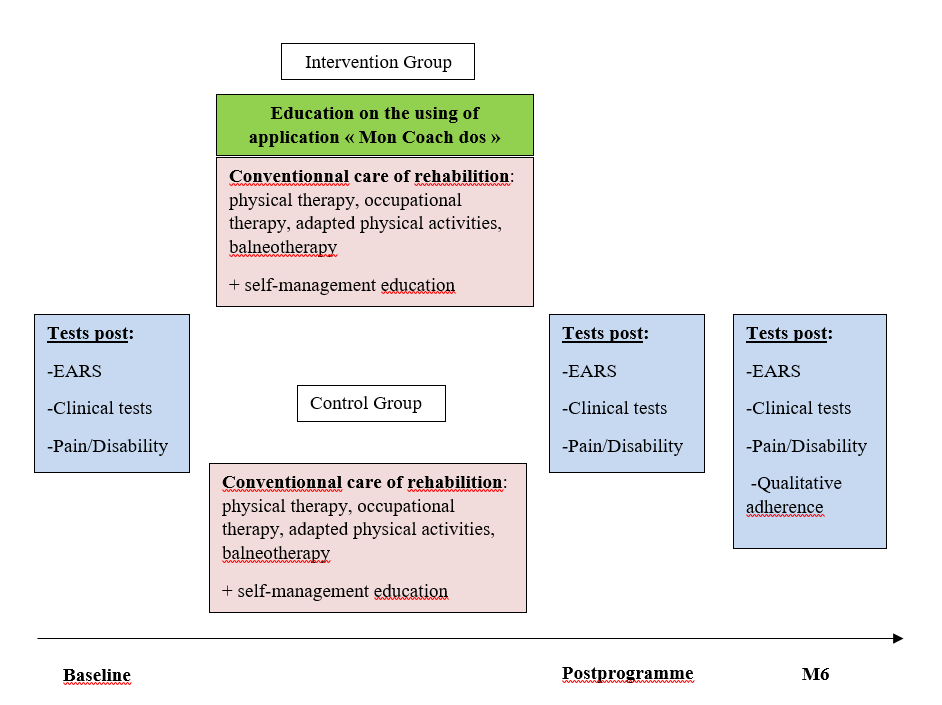

Supplement: Multimedia Appendix 1 [file mhealth-v14-e77736-s001.png]

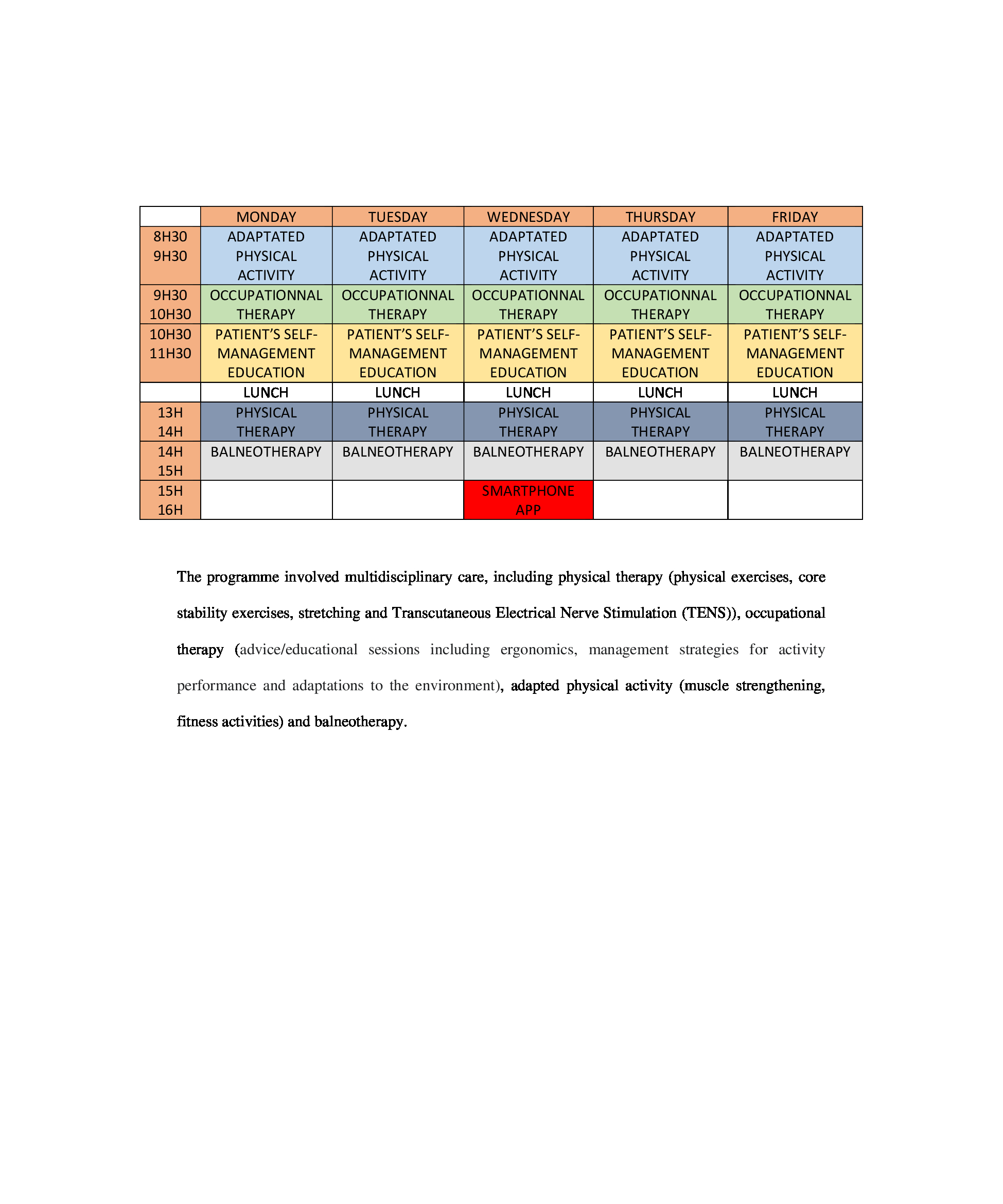

Supplement: Multimedia Appendix 2 [file mhealth-v14-e77736-s002.png]

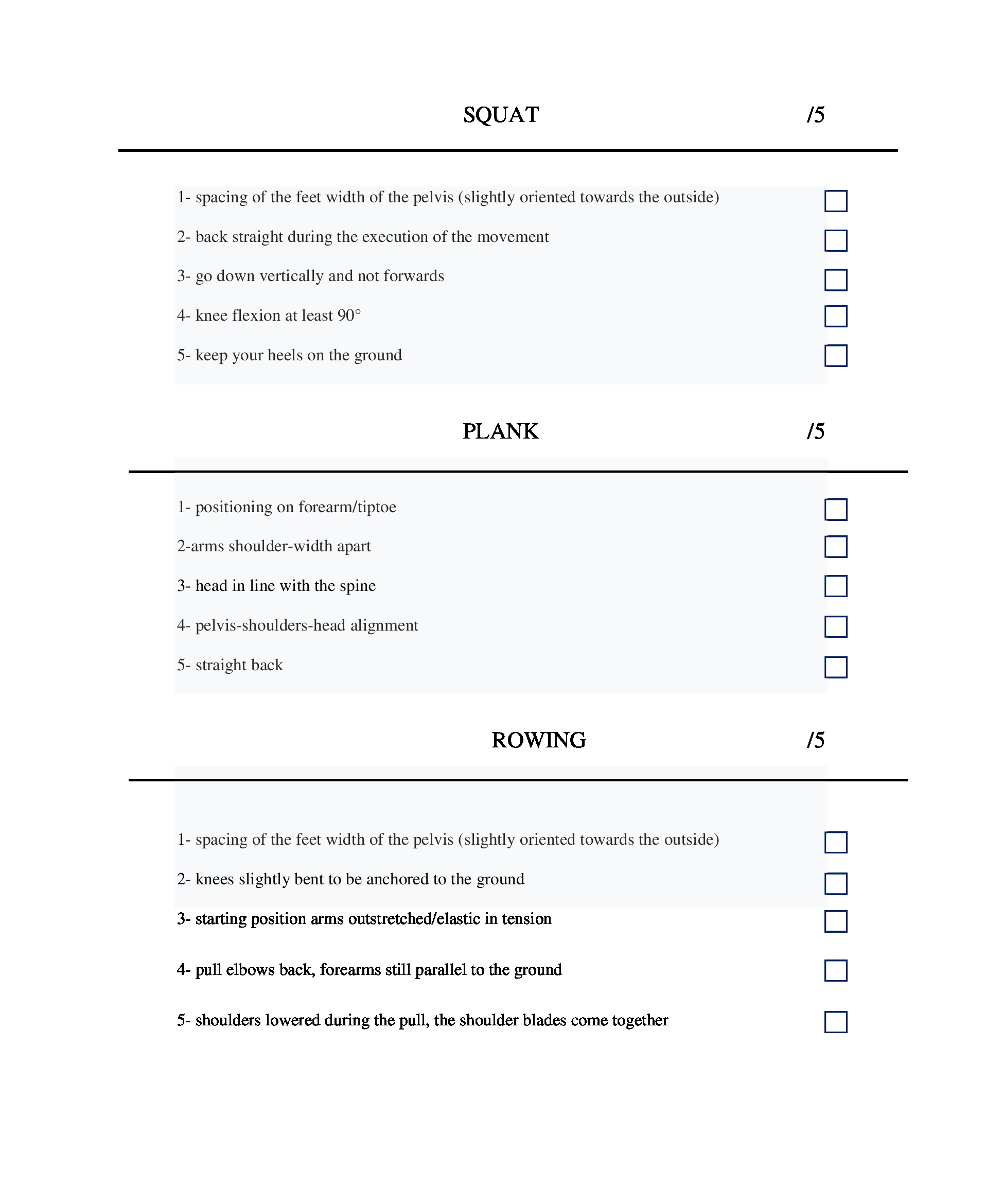

Supplement: Multimedia Appendix 3 [file mhealth-v14-e77736-s003.png]
